# There is no dose–response relationship between allogeneic blood transfusion and healthcare-associated infection: a retrospective cohort study

**DOI:** 10.1186/s13756-021-00928-5

**Published:** 2021-03-29

**Authors:** Yu Lv, Qian Xiang, Jia Lin, Ying Z. Jin, Ying Fang, Hong M. Cai, Qiong D. Wei, Hui Wang, Chen Wang, Jing Chen, Jian Ye, Caixia Xie, Ting L. Li, Yu J. Wu

**Affiliations:** 1 Healthcare-Associated Infection Management Office, Sichuan Academy of Medical Sciences and Sichuan People’s Hospital, Chengdu, 610072 Sichuan People’s Republic of China; 2 Blood Transfusion Department, Sichuan Academy of Medical Sciences and Sichuan People’s Hospital, Chengdu, 610072 Sichuan People’s Republic of China; 3 Healthcare-Associated Infections Control Center, Hospital (T.C.M) Affiliated to Southwest Medical University, LuZhou, Sichuan People’s Republic of China; 4 Department of Nursing, Jianyang People’s Hospital, Jianyang, 641400 Sichuan People’s Republic of China; 5 Nosocomial Infection Management Department, Affiliated Hospital of Sichuan Nursing Vocational College, Chengdu, 610000 Sichuan People’s Republic of China; 6 Department of Nursing, Sichuan Academy of Medical Sciences and Sichuan People’s Hospital, Chengdu, 610072 Sichuan People’s Republic of China; 7 Development Department, Chengdu Yiou Technology Co. LTD, Chengdu, 610000 Sichuan People’s Republic of China

**Keywords:** Healthcare-associated infection, Allogeneic blood transfusion, Restricted cubic spline regression

## Abstract

**Background:**

The association between allogeneic blood transfusion and healthcare-associated infection (HAI) is considered dose-dependent. However, this association may be confounded by transfusion duration, as prolonged hospitalization stay increases the risk of HAI. Also, it is not clear whether specific blood products have different dose–response risks.

**Methods:**

In this retrospective cohort study, a logistic regression was used to identify confounding factors, and the association between specific blood products and HAI were analyzed. Then Cox regression and restricted cubic spline regression was used to visualize the hazard of HAI per transfusion product.

**Results:**

Of 215,338 inpatients observed, 4.16% were transfused with a single component blood product. With regard to these transfused patients, 480 patients (5.36%) developed a HAI during their hospitalization stay. Logistic regression showed that red blood cells (RBCs) transfusion, platelets transfusion and fresh-frozen plasmas (FFPs) transfusion were risk factors for HAI [odds ratio (OR) 1.893, 95% confidence interval (CI) 1.656–2.163; OR 8.903, 95% CI 6.646–11.926 and OR 1.494, 95% CI 1.146–1.949, respectively]. However, restricted cubic spline regression analysis showed that there was no statistically dose–response relationship between different transfusion products and the onset of HAI.

**Conclusions:**

RBCs transfusion, platelets transfusion and FFPs transfusion were associated with HAI, but there was no dose–response relationship between them.

## Introduction

The association between allogeneic blood transfusion (ABT) and healthcare-associated infection (HAI) in hospitalized patients has been described in many studies [1–4]. Whether it is a specific type of infection or a pooled multiple different types of HAI, ABT is considered to increase the risk of HAI. This increased risk of infection is not thought to be due to the direct spread of infectious agents through blood transfusion, but blood transfusions make the body more susceptible to new infections. Allogeneic blood is immunologically active, and the immunosuppressive effects of allogeneic blood known as transfusion-related immunomodulation (TRIM) is considered to be the leading cause of HAI [5]. The underlying mechanism of TRIM is not well understood and is thought to be related to the presence of white blood cells, mediators and bioactive agents [6].

Given the more opportunities the patient will be exposed to immunosuppression when the dose of blood transfusion administered greater, it is a challenge to determine whether increased risk of infection due to the number of transfused units. Previous studies have generally suggested that high doses of blood transfusion increase the risk of infection [7, 8]. However, this association may be confounded by transfusion duration, as prolonged length of hospitalization stay increases the risk of HAI. This is called time bias, which is a confounding factor in HAI-related epidemiological studies that needs to be considered for balance [9]. There are many ways to balance time bias, as mentioned in previous studies, such as matching, propensity scores, and COX regression [10, 11].

Restricted cubic spline (RCS) regression is a powerful tool to characterize a dose–response association between a continuous exposure and an outcome, and to visually check the assumption of linearity of the association. This method has been used in many areas of medical research [12, 13], but it is rarely used in the study of dose–response relationship between HAI and risk factors.

The purpose of this retrospective cohort study was to investigate the dose–response relationship between ABT and HAI. Cox regression was used to adjust for the effect of time bias. Restricted cubic spline regression was used to visualize the hazard of HAI per transfusion product.

## Materials and methods

### Design and subjects

A retrospective cohort study was performed in one of the largest tertiary A-level hospitals in Sichuan Province, China, in which all admissions between the period of 1 January 2017 and 31 December 2018 were included, with the exception of inpatients who were hospitalized within 2 calendar days.

### Cohorts

This study included 4 cohorts, including cohort-RBCs, cohort-FFPs, cohort-cryo and cohort-platelets.

Cohort-RBCs: Inpatients were transfused with RBCs during the study but did not transfuse any other blood product.

Cohort-FFPs: Inpatients were transfused with FFPs during the study but did not transfuse any other blood product.

Cohort-cryo: Inpatients were transfused with cryoprecitation during the study but did not transfuse any other blood product.

Cohort-platelets: Inpatients were transfused with platelets during the study but did not transfuse any other blood product.

### Outcomes

The presence of healthcare-associated infection was the primary outcome of this study.

The diagnostic criteria were the "HAIs Diagnostic Criteria" issued by the Ministry of Health of the People's Republic of China in 2001 [14]. HAIs were defined as infections acquired by inpatients in hospitals, which included infections that occurred during hospitalization and those acquired in hospitals but occurred after discharge. The criteria excluded infections that occurred prior to or at the time of admission.

All reported cases of HAIs were examined by three infectious disease specialists to ensure accurate identification of the infected cases.

### Risk factors for HAI

Potential confounders were selected based on previous literature [15–18], and included age, gender, principal diagnosis, diabetes, hypertension (systolic blood pressure ≥ 140 mmHg and/or diastolic blood pressure ≥ 90 mmHg [19]), chronic obstructive pulmonary disease, hemodialysis, venous catheterization, mechanical ventilation, urinary catheterization, tracheotomy, surgery, ICU admission now or in the past and community infections.

### Data collection

Data was obtained from 3 information systems, including inpatient records system, blood transfusion records system and HAI management information system.

Only ABT given before the onset of HAI were evaluated. Per blood product, a qualitative variable (transfusion yes or no), and 2 quantitative variables (amount of product in units and frequency of transfusion) were generated.

### Statistical analysis

We analyzed the dose–response relationship between blood transfusion components and HAIs using a two-step approach described below. First, we evaluated risk factors associated with HAIs. A logistic regression model with HAIs as the dependent variable was used, and the association between HAIs and RBCs, FFPs, cryoprecitation or platelets were analyzed. Second, Cox regression was performed with the same independent variables as in step 1, and restricted cubic spline regression was used to visualize the hazard of HAI per transfusion product.

Data were summarized using the mean and standard deviation (SD) for normally distributed variables and the median and inter-quartile range (IQR) for non-normally distributed variables. Categorical variables were expressed in absolute numbers and percentages. Statistical analysis of the data was performed using STATA 12.0. Binary outcomes were tested using the χ2 test, and continuous data were compared using Mann–Whitney test or the T-test. *P* values below 0.05 were considered significant.

## Results

### Patient inclusion

**Fig. 1:**
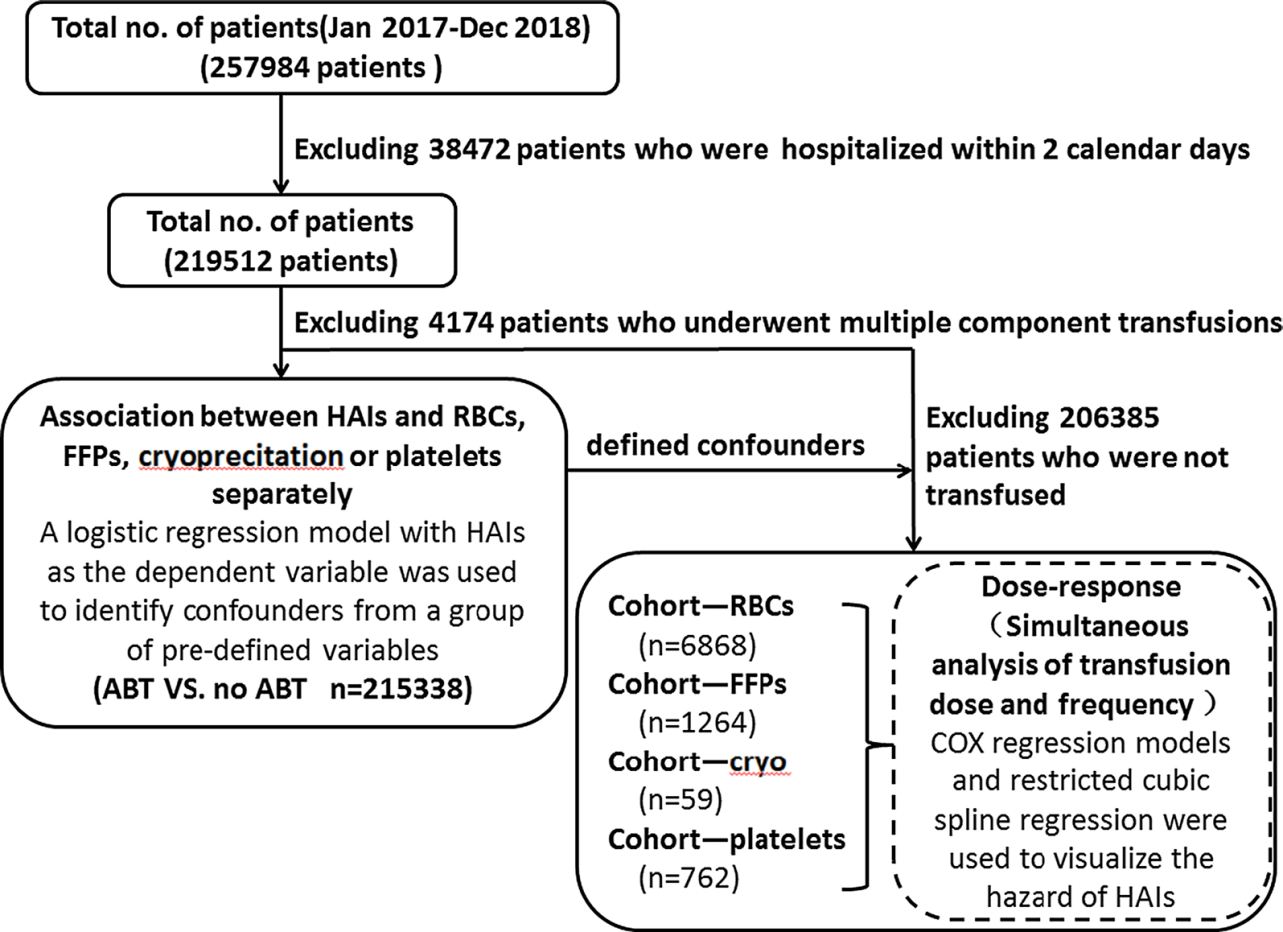
Flow chart of patient inclusion. A total of 257,984 inpatients were admitted to the hospital. After data collation and screening, finally 3079 inpatients with HAI and 212,259 inpatients without HAI were included. This study included 4 cohorts, including cohort-RBCs, cohort-FFPs, cohort-cryo and cohort-platelets

A total of 257,984 inpatients were admitted to the hospital, including 3079 patients with HAI. The first step in this study, 38,472 patients who were hospitalized within 2 calendar days and 4174 patients who underwent multiple component transfusions were excluded. Finally 3079 inpatients with HAI and 212,259 inpatients without HAI were included. In the second step, 206,385 patients who did not undergo transfusions were excluded. Finally 480 inpatients with HAI and 8473 inpatients without HAI were included. See Fig. 1 for details.

**Fig. 2:**
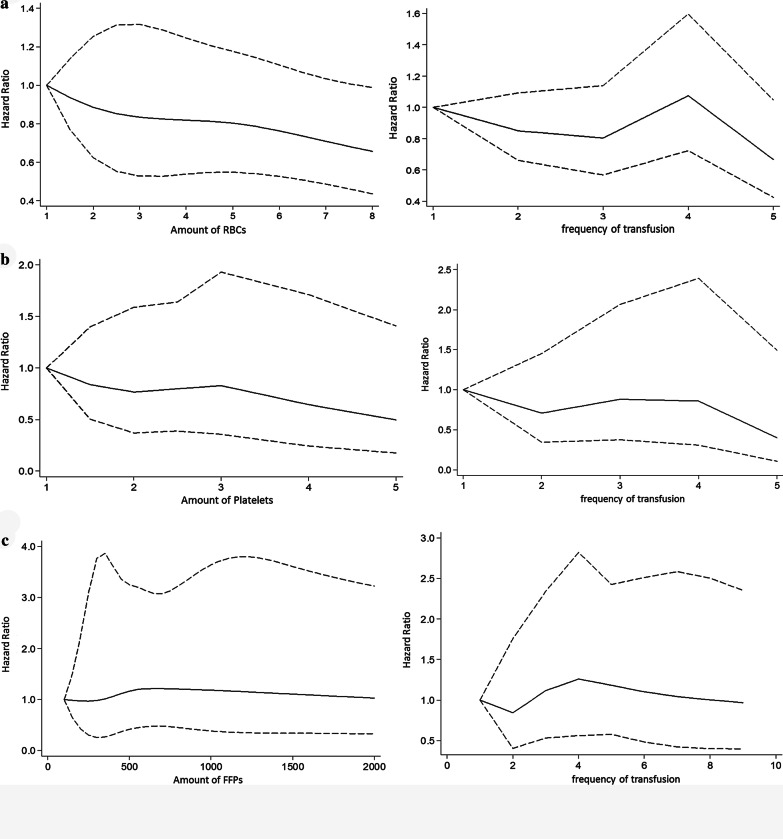
Dose–response relationship between transfusion products and HAI. **a** There was no statistically significant change in the hazard ratio of HAI with the increase in the amount or frequency of RBCs transfusion. **b** There was no statistically significant change in the hazard ratio of HAI with the increase in the amount or frequency of platelets transfusion. **c** There was no statistically significant change in the hazard ratio of HAI with the increase in the amount or frequency of FFPs transfusion

### Characteristics of patients

Of 215,338 inpatients, 4.16% were transfused with a single component blood product. With regard to these transfused patients, 480 patients (5.36%) developed a HAI during their hospitalization stay. Among them, the incidence of HAI caused by platelets transfusion was the highest (8.53%), followed by FFPs transfusion (5.93%), cryoprecitation transfusion (5.08%), RBCs transfusion (4.91%). Characteristics summarized for the patients with HAI and without HAI were listed in Table 1.

**Table 1:** Baseline characteristics

Factors	Overall (N = 215,338)	Non-HAI (N = 212,259)	HAI (N = 3079)	*P*
Age (years ± SD)	51.59 ± 21.74	51.44 ± 21.68	61.71 ± 23.56	< 0.001
ABT [n (%)]				
Non-ABT	206,385 (95.84)	203,786 (96.01)	2599 (84.41)	< 0.001
RBCs	6868 (3.19)	6531 (3.08)	337 (10.95)	
Cryoprecitation	59 (0.03)	56 (0.03)	3 (0.10)	
FFPs	1264 (0.59)	1189 (0.56)	75 (2.44)	
Platelets	762 (0.35)	697 (0.33)	65 (2.11)	
Gender [n (%)]	106,812 (49.60)	104,937 (49.44)	1875 (60.90)	< 0.001
Venous catheterization [n (%)]	13,904 (6.46)	13,189 (6.21)	715 (23.22)	< 0.001
Mechanical ventilation [n (%)]	4888 (2.27)	4290 (2.02)	598 (19.42)	< 0.001
Urinary catheterization [n (%)]	48,195 (22.38)	46,842 (22.07)	1353 (43.94)	< 0.001
Tracheotomy [n (%)]	1421 (0.66)	1194 (0.56)	227 (7.37)	< 0.001
Diabetes [n (%)]	29,428 (13.67)	28,730 (13.54)	698 (22.67)	< 0.001
Hypertension [n (%)]	52,713 (24.48)	51,392 (24.21)	1321 (42.90)	< 0.001
Chronic obstructive pulmonary disease [n (%)]	7902 (3.67)	7545 (3.55)	357 (11.59)	< 0.001
ICU admission now or in the past [n (%)]	12,997 (6.04)	12,159 (5.73)	838 (27.22)	< 0.001
Hemodialysis [n (%)]	451 (0.21)	428 (0.20)	23 (0.75)	< 0.001
Surgery [n (%)]	110,353 (51.25)	108,675 (51.20)	1678 (54.50)	< 0.001
Community infections [n (%)]	28,396 (13.19)	27,808 (13.10)	588 (19.10)	< 0.001
Principal diagnosis (ICU-10 code) [n (%)]				
Certain infectious diseases and parasites(A00-B99)	6561 (3.05)	6404 (3.02)	157 (5.10)	< 0.001
Tumor (C00-D48)	30,975 (14.38)	30,505 (14.37)	470 (15.26)	
Blood and hematopoietic diseases and certain diseases involving immune mechanisms (D50-D89)	1735 (0.81)	1710 (0.81)	25 (0.81)	
Endocrine, nutritional, and metabolic diseases (E00-E90)	5453 (2.53)	5355 (2.52)	98 (3.18)	
Mental and behavioral disorders (F00-F99)	3717 (1.73)	3668 (1.73)	49 (1.59)	
Nervous system diseases (G00-G99)	7870 (3.65)	7699 (3.63)	171 (5.55)	
Eye and appendage diseases (H00-H59)	6051 (2.81)	6050 (2.85)	1 (0.03)	
Ear and mastoid diseases (H60-H95)	2092 (0.97)	2086 (0.98)	6 (0.19)	
Circulatory diseases (I00-I99)	27,682 (12.86)	27,109 (12.77)	573 (18.61)	
Respiratory diseases (J00-J99)	17,848 (8.29)	17,287 (8.14)	561 (18.22)	
Digestive diseases (K00-K93)	26,575 (12.34)	26,383 (12.43)	192 (6.24)	
Skin and subcutaneous tissue diseases (L00-L99)	3201 (1.49)	3176 (1.50)	25 (0.81)	
Musculoskeletal system and connective tissue diseases (M00-M99)	11,295 (5.25)	11,124 (5.24)	171 (5.55)	
Genitourinary diseases (N00-N99)	15,174 (7.05)	15,010 (7.07)	164 (5.33)	
Pregnancy, childbirth, and puerperium (O00-O99)	11,294 (5.24)	11,256 (5.30)	38 (1.23)	
Diseases that originated in the perinatal period (P00-P96)	1177 (0.55)	1137 (0.54)	40 (1.30)	
Congenital malformations, deformation, and chromosomal abnormalities (Q00-Q99)	3595 (1.67)	3577 (1.69)	18 (0.58)	
Abnormal symptoms, signs, clinical and laboratory results, and cannot be classified in other categories (R00-R99)	2663 (1.24)	2652 (1.25)	11 (0.36)	
Injury, poisoning and other external pathogenic factors (S00-T98)	9192 (4.27)	9058 (4.27)	134 (4.35)	
External causes of illness and death (V01-V98)	21,188 (9.84)	21,013 (9.90)	175 (5.68)	

The median RBCs amount and transfusion frequency of patients who developed a HAI were longer compared to patients who did not develop a HAI [2 (IQR 2–3) vs. 2 (IQR 2–4) units and 1 (IQR 1–2) vs. 1 (IQR 1–3) times, respectively]. The median platelets amount of patients who developed a HAI was longer compared to patients who did not develop a HAI [1 (IQR 1–2) vs. 1 (IQR 1–3) units, respectively]. Table 2 shows these details.

**Table 2:** Transfusion characteristics

Patients	Overall	Non-HAI	HAI	P
RBCs				
No	6868	6531	337	
Amount of transfusion [median (IQR)]	2 (2–4)	2 (2–3)	2 (2–4)	< 0.001
Frequency of transfusion [median (IQR)]	1 (1–2)	1 (1–2)	1 (1–3)	0.013
Cryoprecitation				
No	59	56	3	
Amount of transfusion [median (IQR)]	1 (1–1)	1 (1–1)	1 (1–1)	0.521
Frequency of transfusion [median (IQR)]	1 (1–1)	1 (1–1)	1 (1–1)	0.547
FFPs				
No	1264	1189	75	
Amount of transfusion [median (IQR)]	350 (200–600)	350 (200–600)	400 (200–750)	0.315
Frequency of transfusion [median (IQR)]	1 (1–3)	1 (1–3)	1 (1–3)	0.952
Platelets				
No	762	697	65	
Amount of transfusion [median (IQR)]	1 (1–2)	1 (1–2)	1 (1–3)	0.010
Frequency of transfusion [median (IQR)]	1 (1–2)	1 (1–2)	1 (1–2)	0.060
Time between (first) transfusion and HAI (days)				
RBCs			9 (6–15)	
Cryoprecitation			8 (4–14)	
FFPs			10 (6–16)	
Platelets			8 (4–13)	

### Logistic regression

Logistic regression identified RBCs transfusion, FFPs transfusion, platelets transfusion, age, gender, principal diagnosis, diabetes, hypertension, chronic obstructive pulmonary disease, hemodialysis, venous catheterization, mechanical ventilation, urinary catheterization, tracheotomy, surgery, ICU admission now or in the past and community infections as independent predictors for HAI. The details are shown in Table 3.

**Table 3:** Equation parameters of confounding factors affecting HAI

Covariate	OR	95% C.I. for OR		*P*
		Lower	Upper	
Constant	–	–	–	< 0.001
ABT				
RBCs	1.893	1.656	2.163	< 0.001
Cryoprecitation	2.459	0.730	8.290	0.147
FFPs	1.494	1.146	1.949	0.003
Platelets	8.903	6.646	11.926	< 0.001
Gender	1.307	1.210	1.411	< 0.001
Venous catheterization	1.897	1.693	2.126	< 0.001
Mechanical ventilation	2.185	1.895	2.519	< 0.001
Urinary catheterization	1.953	1.776	2.149	< 0.001
Tracheotomy	5.473	4.607	6.501	< 0.001
Diabetes	1.231	1.117	1.356	< 0.001
Hypertension	1.475	1.350	1.612	< 0.001
Chronic obstructive pulmonary disease	1.404	1.220	1.615	< 0.001
Age	1.015	1.013	1.018	< 0.001
ICU admission now or in the past	1.422	1.255	1.610	< 0.001
Hemodialysis	2.110	1.337	3.329	0.001
Surgery	1.107	1.022	1.198	0.012
Community infections	1.218	1.084	1.368	0.001
Principal diagnosis (ICU-10 code)				
Certain infectious diseases and parasites(A00-B99)	–	–	–	< 0.001
Tumor (C00-D48)	0.439	0.361	0.534	< 0.001
Blood and hematopoietic diseases and certain diseases involving immune mechanisms (D50-D89)	0.523	0.334	0.820	0.005
Endocrine, nutritional, and metabolic diseases (E00-E90)	0.692	0.529	0.904	0.007
Mental and behavioral disorders (F00-F99)	0.766	0.550	1.067	0.115
Nervous system diseases (G00-G99)	0.841	0.667	1.061	0.145
Eye and appendage diseases (H00-H59)	0.009	0.001	0.067	< 0.001
Ear and mastoid diseases (H60-H95)	0.199	0.088	0.452	< 0.001
Circulatory diseases (I00-I99)	0.573	0.473	0.695	< 0.001
Respiratory diseases (J00-J99)	0.763	0.625	0.931	0.008
Digestive diseases (K00-K93)	0.331	0.266	0.412	< 0.001
Skin and subcutaneous tissue diseases (L00-L99)	0.493	0.321	0.758	0.001
Musculoskeletal system and connective tissue diseases (M00-M99)	0.750	0.597	0.941	0.013
Genitourinary diseases (N00-N99)	0.414	0.328	0.522	< 0.001
Pregnancy, childbirth, and puerperium (O00-O99)	0.244	0.168	0.353	< 0.001
Diseases that originated in the perinatal period (P00-P96)	2.595	1.753	3.843	< 0.001
Congenital malformations, deformation, and chromosomal abnormalities (Q00-Q99)	0.263	0.160	0.434	< 0.001
Abnormal symptoms, signs, clinical and laboratory results, and cannot be classified in other categories (R00-R99)	0.222	0.120	0.413	< 0.001
Injury, poisoning and other external pathogenic factors (S00-T98)	0.432	0.338	0.553	< 0.001
External causes of illness and death (V01-V98)	0.476	0.378	0.598	< 0.001

### Restricted cubic spline regression

Restricted cubic spline regression analysis showed that there was no statistically dose–response relationship between different transfusion products and the onset of HAI, as Fig. 2 shows. Because cryoprecitation has not been found to be an independent risk factor for HAI in logistic regression, it was not included in this section.

## Discussion

This study found that RBCs transfusion, FFPs transfusion and platelets transfusion were independent risk factors for HAI, while cryoprecitation was not. A restrictive transfusion strategy may be an important first step to infection prevention. A previous meta-analysis concluded that a restrictive transfusion strategy was associated with a reduced risk of HAI when compared with a liberal transfusion strategy [20]. As such, clinicians need to actively seek alternatives to transfusion before evaluating the risks and benefits of transfusion in individual patients [21]. Our study also found that the differences in HAI risk caused by different blood products are significant. Obvious differences in HAI risk may have an impact on the definition of HAI susceptible population and the formulation of prevention and control strategies. However, even with restrictive transfusion, adherence to adoption of certain blood products for on-demand treatment is an important second step in preventing HAI.

In our study, platelets transfusion had the highest OR value, which was much greater than any other risk factor. Like previous studies [1, 22, 23], the increased HAI risk due to platelets transfusion was highlighted, and platelets transfusion was considered to be the most important blood products associated with HAI in ours study. In line with a prospective multicenter cohort study from Netherlands [24], it was unlikely that RBCs contributed to the risk of infections when administered together with platelets. Although the effectiveness of platelet transfusions to prevent bleeding had been affirmed at a platelet count of 10 × 10^9^/L in acute leukemia patients [25], it lacked data support in other patients. There is a clear indication for platelets transfusion only in hematology patients [26, 27]. Given the association between platelet transfusion and increased HAI risk, we believe that a risk benefit analysis should be conducted in relation to continued platelet transfusion.

We found that there was no dose–response relationship between ABT and HAI. Regardless of the type of transfusion product, the hazard ratio of HAI was not linearly related to their transfusion units or frequency. This is in contrast to other studies [28–30], which demonstrated an association between transfusion and complications was dose-dependent. However, these dose–response relationships are not entirely credible, because the division of dose groups in these studies is completely different, without any reference. We believe that potential classification errors are likely to lead to biased results. Similarly, this difference might be explained by the use of different statistical analysis methods. Our results were based on both Cox regression and restricted cubic spline regression, whereas in previous studies, the length of time from the first blood transfusion to infection was ignored, resulting in a large time bias. In addition, different preparation methods and storage schemes might also be the cause of this difference. Consistent with our findings, a study from the United States found that there did not seem to be a dose–response relationship between the number of blood units transfused and any of the postoperative infections [31].

Optimal use of blood transfusions should involve administering enough to maximize clinical outcomes while avoiding unnecessary exposure to the HAI risk [32]. To achieve this almost impossible ideal state, more evidence-based guidelines are needed to provide clinicians with feasible methods. Our findings did not appear to be clinically relevant, but could eliminate concerns about doses during blood transfusions. If blood transfusion was unavoidable according to the patient's condition, methods to administer enough doses as well as to increase the effectiveness of each unit transfused would greatly benefit patients, by avoiding and preventing the occurrence of HAI. The practical significance of our findings might be of reference value in HAI monitoring and infection-related risk assessment. ABT in our study was an associated and apparently significant risk factor for the development of HAI, but it was not defined as a risk factor in China’s nosocomial infection surveillance guideline [33], leading to the neglect of ABT in many previous studies [34–36]. Given that ABT is such a significant and frequently overlooked risk factor for HAI, it is necessary to include ABT as invasive procedures and antimicrobial resistance into the daily targeted surveillance of HAI. We recommend that future studies of blood transfusion management should include HAI as a patient outcome so that transfusion-related immunomodulation can be more widely estimated and evaluated.

Previous studies have indicated that the storage time of blood products may be central to their immune modulatory effect. Khan et al. found that soluble CD40 ligand accumulated in stored blood components, primes neutrophils through CD40, and was a potential cofactor in the development of transfusion-related acute lung injury [37]. Kristiansson et al. found that stored RBCs had high levels of cytokines but that the specific cytokine levels varied as a function of the age of the blood. Interleukin-1 levels, for instance, increased with time, whereas interleukin-6 levels remained elevated throughout the duration of RBCs storage [38]. However, the storage time of blood products was not analyzed in our study because the transfusion of older blood products compared with fresher blood products was not associated to worse clinical complication in recent large trial studies [39, 40].

We acknowledge several limitations in our study. Most importantly, transfusion-related immunomodulation varied across specific types of infections. Our study has pooled multiple different types of HAI. Thus, the potential dose–response relationship between a specific type of infection and ABT may be ignored. Further studies are needed to distinguish the types of infection, to further explore the dose–response relationship between specific types of infection and ABT. Furthermore our study was retrospective and only involved the analysis of a hospital. Larger multi-center randomized studies should be conducted to validate our findings.

## Conclusions

Our study found that RBCs transfusion, platelets transfusion and FFPs transfusion were associated with HAI, but there was no dose–response relationship between them. If blood transfusion was unavoidable, we recommend administering enough doses to maximize clinical benefit.

## Data Availability

All data generated and analyzed during this study are included in this article.
